# AIRE acetylation and deacetylation: effect on protein stability and transactivation activity

**DOI:** 10.1186/s12929-014-0085-z

**Published:** 2014-08-27

**Authors:** Federica Incani, Maria Luisa Serra, Alessandra Meloni, Carla Cossu, Luisella Saba, Tiziana Cabras, Irene Messana, Maria Cristina Rosatelli

**Affiliations:** Dipartimento di Sanità Pubblica, Medicina Clinica e Molecolare, Unità di Ricerca di Scienze Biomediche e Biotecnologie, Università degli Studi di Cagliari, via Jenner s/n, Cagliari, Italy; Istituto di Ricerca Genetica e Biomedica, Consiglio Nazionale delle Ricerche, Cagliari, Italy; Dipartimento di Scienze della Vita e dell’Ambiente, Sezione di Biochimica, Università degli Studi di Cagliari, Cagliari, Italy

**Keywords:** Autoimmune regulator, Acetylation, Deacetylation, Mass spectrometry, HDAC1-HDAC2/SIN3A complex

## Abstract

**Background:**

The AIRE protein plays a remarkable role as a regulator of central tolerance by controlling the promiscuous expression of tissue-specific antigens in thymic medullary epithelial cells. Defects in AIRE gene cause the autoimmune polyendocrinopathy- candidiasis-ectodermal dystrophy, a rare disease frequent in Iranian Jews, Finns, and Sardinian population.

AIRE protein is primarily known as a transcriptional regulator and is capable of interacting with numerous proteins. The first characterized partner of AIRE is the ubiquitous transcription factor CREB-binding protein (CBP), which regulates DNA transcription through the acetylation and deacetylation of histones. More recently, the role of p300 in AIRE acetylation, which could influence the selection of AIRE activated genes, has been described.

**Results:**

In this study, we have precisely mapped, by mass spectrometry experiments, the sites of protein acetylation and, by mutagenesis assays, we have described a set of acetylated lysines as being crucial in influencing the subcellular localization of AIRE. Furthermore, we have also determined that the de-acetyltransferase enzymes HDAC1-2 are involved in the lysine de-acetylation of AIRE.

**Conclusions:**

On the basis of our results and those reported in literature, we propose a model in which lysines acetylation increases the stability of AIRE in the nucleus. In addition, we observed that the interaction of AIRE with deacetylases complexes inhibits its transcriptional activity and is probably responsible for the instability of AIRE, which becomes more susceptible to degradation in the proteasome.

## Background

Autoimmune polyendocrinopathy candidiasis ectodermal dystrophy (APECED or APS-1) is a rare monogenic autoimmune disease caused by mutations in the autoimmune regulator (AIRE) gene. The clinical manifestation in patients with APECED includes concurrent organ-specific autoimmune disorders, due to the loss of self-tolerance to certain antigens. The AIRE protein induces the expression of a battery of peripheral tissue self-antigens (PTAs) in thymic stromal cells, thus promoting the clonal deletion of differentiating T cells that recognize them [1]. For this reason, the study of the function of AIRE protein represents an excellent biological model to dissect the mechanisms of autoimmunity.

AIRE protein is primarily known as a transcriptional regulator which functions as an activator or repressor, depending on the cellular context in which the protein is functionally active. AIRE is mainly localized in the nucleus, where it assembles in aggregates called nuclear bodies. It is characterized by several domains involved in nuclear transport, in transcriptional activation, in DNA binding, in E3 ubiquitin ligase activity and in transcriptional repression. In particular, the DNA binding domain, also called the SAND domain (**S**P100, **A**IRE, **N**ucP41/75 and **D**EAF-1), is a typical feature of proteins involved in chromatin-mediated transcriptional regulation. The four LXXLL domains, placed both in the carboxyl and amino terminal regions, represent potential binding sites for nuclear receptors and are involved in numerous protein-protein interactions related to various phases of its transcriptional regulator activity. In particular, the last LXXLL motif and the flanking PXXPXP sequence are essential for the transactivation capacity of AIRE [2]. This finding is supported by the presence in this region of an APECED causing mutation [3].

Two Plant Homeo Domain-type zinc fingers (PHD) which are common in all eukaryotes and are involved in chromatin-mediated transcriptional regulation, are localized in the C-terminal region [4]. AIRE PHDs are multifunctional domains, with transactivation and repression activity [2]. The N-terminal region of AIRE was initially described as an HSR (Homogenously Staining Region) domain; however, a recent structural reanalysis revealed a six-helix structure with high similarity to a caspase-recruitment domain (CARD). The CARD domain is implicated in homodimerization of AIRE and in heterodimerization with other proteins that function either in inflammation or apoptosis [5]. Furthermore, within the HSR domain, the presence of a potential nuclear export signal (NES) has been described [6]. More recent reports have shown that several APECED-causing mutations within the CARD or SAND domain of AIRE also decrease its transactivation ability, indicating that all of the main AIRE domains are involved in transcriptional activation, although the effect of these mutations is extremely variable [5,7,8]. Finally, in addition to the HSR/CARD domain, the AIRE protein also includes a nuclear localization signal (NLS) [9].

Recent proteomic studies [1] have led to the identification of several proteins that interact with AIRE, grouped into functional classes i.e., nuclear proteins, proteins that bind chromatin or are involved in transcription or, finally, in RNA processing. The great variety of protein activities characterized so far shows the broad and mostly unclarified range of cellular processes and pathways in which AIRE could possibly be involved. The ubiquitous transcription factor CREB-binding protein (CBP), which regulates DNA transcription through the acetylation and deacetylation of histones, was the first characterized partner of AIRE [10]. Several studies have shown that AIRE-CBP interactions occur in nuclear bodies and increase the expression of its target genes [5,8,11].

More recently, Saare et al. [12] described the role of p300 in AIRE acetylation, which could influence the selection of AIRE activated genes. In particular, by mass spectrometry experiments, the sites of protein acetylation were precisely mapped and, by mutagenesis assays, a set of acetylated lysines, placed in the SAND domain, was described as being crucial in influencing the selection of genes regulated by AIRE [12]. Other studies have shown that acetylation enhances nuclear/cytoplasmic shuttling of protein targets and, in some cases, it is essential for this dynamic action. For example, POP-1 requires the acetylation of certain lysines to gain entry to the nuclear compartment [13]**,** whereas the acetylation of proteins such as Max, c-Abl and RECQL4 increases their cytoplasmic localization [14-17].

The exact mechanisms and the specific aminoacid residues involved in the nuclear shuttling of AIRE have been partially defined [18]. For this reason, one of the aims of our study was to investigate, by mutagenesis assays and immunofluorescence imaging, the role of the N-terminal domain in the subcellular localization of AIRE. Our results demonstrated that the acetylation of lysine's 102-111-131-133 is involved in the correct cellular positioning of AIRE, as the substitution of these residues with arginines abolishes its nuclear localization. Since acetylation is a reversible process, once it was determined that AIRE is deacetylated, we explored the possible role of the HDAC1/HDAC2/SIN3A complex in the deacetylation process. By co-immunoprecipitation assays, we found that AIRE directly interacts with the HDAC1/HDAC2/SIN3A complex and that AIRE deacetylation results in a decreased AIRE transactivating activity. These results suggest that the activity of AIRE is finely regulated by its alternating acetylation and deacetylation status.

On the basis of our data and those reported in literature, we propose a model in which lysine acetylation increases the stability of AIRE in the nucleus. Furthermore, as lysines could also be a target for ubiquitination and subsequent proteolysis, their deacetylation could be the first step before degradation. Consequently, acetylation could somehow protect AIRE from degradation when its transcriptional activity is required in the nucleus.

## Methods

### Plasmids

#### Plasmids used in the acetylation assay

For MS/MS and for the co-immunoprecipitation assays, full-length AIRE was cloned into EcoRI and NotI restriction sites of the plasmid pEF5HA, kindly provided by Nunzio Bottini (University of Southern California, Los Angeles, CA). The pEFHA vector allows the expression of constructs in fusion with an N-terminal HA tag, under control of the EF alpha promoter.

#### Plasmids used in deacetylation assay

AIRE cDNA (nucleotides corresponding to aminoacids 1–545) was amplified by polymerase chain reaction using primers carrying a EcoRV restriction site and cloned into HaloTag pHT2vector (Promega). The human full-length HDAC1 and HDAC2 cDNAs cloned into pCMV-XL5 plasmid were purchased from OriGene (Rockville, MD).

#### Plasmids used in fluorescence microscopy assay

The full-length AIRE cDNA was amplified and cloned into the EcoRV restriction site of the Monster Green Fluorescent Protein (phMGFP)-expression vector (Promega, Madison WI).

The mutated AIRE constructs Lys 102Arg-Lys111Arg-Lys131Arg-Lys133Arg and Lys102Gln-Lys111Gln-Lys131Gln-Lys133Gln were obtained by site-specific mutagenesis of the phMGFP-AIRE full-length construct.

#### Plasmids used in luciferase assay

The cDNA encoding AIRE (nucleotides corresponding to aminoacids1-545) was amplified with primers carrying an XbaI restriction site and cloned into the mammalian expression vector pBIND which contains a GAL4 DNA-binding domain (Promega).

### Antibodies

The anti-HA monoclonal antibody (clone 16B12) and was purchased from Covance (Berkeley, CA) Polyclonal anti-HDAC1, HDAC2 and monoclonal mSIN3A (G.-11), sc-5299 antibodies were purchased from Santa Cruz Biotech (Santa Cruz, CA). The acetylated lysine polyclonal antibody (#9441) was purchased from Cell Signaling (Euroclone, Milan, Italy) and the anti-HaloTag® polyclonal antibody was purchased from Promega Corporation (Madison, WI). The anti-actin antibody was purchased from Sigma.

The anti-mouse and anti-rabbit secondary antibodies for western blotting were purchased from GE Healthcare (Piscataway, NJ).

### Site-directed mutagenesis

PCR reactions were carried out using the QuickChange II site-directed mutagenesis kit (Agilent Technologies), according to manufacturer’s instructions. The sense and anti-sense oligonucleotides used are listed in Table 1. After PCR, the parental supercoiled dsDNA was digested by adding 1 μL of Dpn1 restriction enzyme (10 U/μL) and incubating at 37°C for 1 hour. The DpnI digested dsDNA was transformed into *E. coli* DH5α competent cells. The presence of each mutation was confirmed by direct sequencing of selected colonies.

**Table 1:** **Primers used in the present study**

**Construct**	**Oligonucleotidesequence (5’-3’)**
AIRE Lys102Gln	Fprimer: CTGGACAGCTTCCCCCAAGATGTGGACCTCAGCCA
	Rprimer: TGGCTGAGGTCCACATCTTGGGGGAAGCTGTCCAG
AIRE Lys102Arg	Fprimer: CTGGACAGCTTCCCCAGAGATGTGGACCTCAGCCA
	Rprimer: TGGCTGAGGTCCACATCTCTGGGGAAGCTGTCCAG
AIRE Lys111Gln	F-primer: CTCAGCCAGCCCCGGCAGGGGAGGAAGCCCCCG
	R-primer: CGGGGGCTTCCTCCCCTGCCGGGGCTGGCTGAG
AIRE Lys111Arg	F-primer: CTCAGCCAGCCCCGGAGGGGGAGGAAGCCCCCG
	R-primer: CGGGGGCTTCCTCCCCCTCCGGGGCTGGCTGAG
AIRE Lys131-133Gln	F-primer: CTCCCCACCCAGAGGCAGGCCTCAGAAGAGGCT
	R-primer: AGCCTCTTCTGAGGCCTGCCTCTGGGTGGGGAG
AIRE Lys131-133Arg	F-primer: CTCCCCACCAGGAGGAGGGCCTCAGAAGAGGCT
	R-primer: AGCCTCTTCTGAGGCCCTCCTCCTGGTGGGGAG

*Abbreviations*: *F-primer* Forward primer, *R-primer* Reverse primer.

### Cell culture and transient transfections

COS-1 cells were grown in DMEM (high glucose), supplemented with 10% FCS, 100 units/mL of penicillin, 100 μg/mL streptomycin and 2 mM glutamine (Invitrogen), at 37°C and 5% CO_2_. For the transactivation assay, COS-1 cells were seeded into 35 mm tissue culture plates, at a density of 2 × 10^5^cells/plate. All transfections were performed by the Lipofectamine 2000 method according to the manufacturer’s instructions (Invitrogen). Each experiment was repeated at least three times, and, within each experiment, the transfections were performed in duplicate.

### Luciferase report assay

For the deacetylation assay, COS-1 cells were transfected with 2.5 μg of GAL4-DNA-BD AIRE fusion protein alone, and with pCMV-XL5-HDAC1 and pCMV-XL5-HDAC2 together and separately, at a concentration of 300 ng each, in the presence of 2.5 μg of the reporter plasmid pG5luc (Promega) wich contains a luciferase gene, using Lipofectamine method. Forty-eight hours after transfection, cells were harvested and lysed with passive lysis buffer (Promega). Luciferase assays were performed on cell lysates using the Dual Luciferase Reporter Assay kit from Promega (Madison, WI), following the manufacturer’s instructions. Luminescence was measured with a Microlumat LB 96P luminometer (Berthold).

For deacetylation inhibition studies, 98% sodium butyrate (NaBu; Sigma) was added 4 h after transfection at a final concentration of 10 mM and incubated for 24 h.

### Microscope analysis for nuclear dot visualization

COS-1 cells transfected with the phMGFP-AIRE wt and phMGFP-AIRE mutant constructs and seeded on culture slides were fixed with MeOH for 5 min on ice and with EtOH at room temperature for 10 min, permeabilized with PBS containing 1% Triton X-100 for 10 min at room temp, and blocked for 45 min with 8% bovine serum albumin (BSA) in PBS. Nuclei were stained, for 5 min at room temperature, with 2 μg/mL 4,6-diamidino 2-phenylindole (DAPI; Invitrogen) diluted 1:1000 in PBS. Culture slides were finally mounted with a coverglass. Cells were examined by a Leica DM6000B microscope (Leica Microsystems GmbH, Wetzlar, Germany). Images were acquired using Leica Laser Microdissection LMD software (Leica Microsystems, version 6.6.0) and a triple band pass filter (Leica Microsystems, B/G/R fluorescence filter).

### Immunoprecipitation

For every immunoprecipitation experiment, transfected COS-1 cells (~10^6^) were scraped and washed twice with ice-cold PBS. Pellets were lysed on ice in 0.5 mL of lysis buffer containing 50 mMTris-HCl, pH 7.5, 50 mMNaCl, 0.2% Nonidet P-40, 5 μM ZnCl_2_, 30 mM Na_4_P_2_O_7_, 50 mMNaF, 2 mM sodium orthovanadate, 1 mM phenylmethylsulfonyl fluoride (PMSF) and a protease inhibitor tablet (Roche). Cell lysates were centrifuged at 12,000 g for 20 minutes at 4°C and the supernatants (500–700 μg of protein extracts for each experiment) were pre-clarified with 50 μL of 50% protein G Sepharose 4B resin (GE Healthcare) for 4 hours at 4°C. After centrifugation, 3 μL of the antibody were added to the supernatant, followed by agitation overnight at 4°C. A further 50 μL of 50% protein G Sepharose 4B resin were added to each sample and incubated with gentle rotation at 4°C for 4 h, to recover protein complexes. The resin was washed four times with PBS containing 0.2% NP40, and the immune complexes were released from the resin by boiling in 30 μl of 2x Laemmli buffer (100 mM Tris-HCl pH 6.8, 200 mM DTT, 0.2% bromophenol blue, 20% glycerol) for 5 min.

The proteins that immunoprecipitated with the anti-HALO antibody were used for the deacetylation assay, while the proteins that immunoprecipitated with anti-HA were used for mass-spectrometry analysis. The anti-mSIN3A (1:2000) was used to detect the direct interaction with AIRE-HA

### Western blotting

The immunoprecipitated samples were analyzed by 10% SDS-PAGE and transferred to a nitrocellulose membrane (Hybond ECL; GE Healthcare). After transfer, the filters were blocked with 5% non-fat dry milk in TBS-T buffer (100mMTris-HCl pH7.5, 150mMNaCl, 0.1% Tween 20) for 4 hours at 4°C. Primary and secondary antibodies were incubated in 5% non-fat dry milk in TBST buffer, for 18 h and 2 h, respectively. Signals were detected using the ECL Plus Chemiluminescence method (GE Healthcare) and captured on Hyperfilm ECL high performance chemiluminescence film (GE Healthcare). Anti-acetylated lysine (1:2000) and anti-Halo tag (1:5000) antibodies were used to establish the presence of acetylated AIRE. The deacetylation assays were performed by Anti HDAC1 and HDAC2 antibodies (1:10000). Anti-mSIN3A (1:2000) was used to detect the interaction with AIRE-HA. The following secondary antibodies were used: anti-mouse (1:5000) for AIRE-HA, HDAC1 and HDAC2, and anti-goat (1:100000) for AIRE-Halo detection.

### Proteolytic digestion and mass spectrometry analysis

Immunoprecipitated HA-AIRE was separated by 10% SDS-PAGE. The gel was rinsed with 100 ml of ultrapure water three times, then stained with Simply Blue SafeStain (Invitrogen) for 1 h at room temperature with gentle shaking. The band corresponding to HA-AIRE was cut and the excised gel slice was incubated in 50% H_2_O/MeOH with 5% acetic acid for 3–4 hours with gentle vortexing; the solution was refreshed several times until the dye was extracted from the gel. Gel slices, each containing from 0.5 to 1 μg of protein, were then submitted to the in-gel trypsin digestion protocol according to Link et al. [19], and the recovered tryptic peptides were pooled. Alternatively, HA-AIRE was digested with endoprotease Glu-C after western blotting. Nitrocellulose (NC) bands containing ca. 1 μg of the intact protein were incubated for 30 min at room temperature in acetone (90 μL acetone/4 mm^2^ NC) in order to dissolve completely the NC and precipitate the protein. After centrifugation, the supernatant was discharged and the precipitate was solubilized in 3% aqueous formic acid (FA) and 2% acetonitrile. The solution was then lyophilized, the powder dissolved in 100 mM ammonium bicarbonate pH 8.0 and digested at 37°C with endoprotease Glu-C (1:50, enzyme/protein) overnight.

The tryptic peptides and the Glu-C peptides were separately lyophilized, resuspended in 50 μL of 0.1% FA and analyzed both by high resolution RP-HPLC-ESI-MS/MS and MALDI-TOF-MS. High-resolution HPLC-ESI-MS/MS experiments were performed using an Ultimate 3000 Micro HPLC apparatus (Dionex, Sunnyvale, CA) equipped with an FLM-3000-Flow manager module coupled to an LTQ Orbitrap XL apparatus (ThermoFisher). A Zorbax 300SB-C18 column (3.5 μm particle diameter; column dimension 1.0 × 150 mm) was used. The eluents were: (A) 0.05% (v/v) aqueous TFA and (B) 0.05% (v/v) TFA in acetonitrile. The applied gradient was: 0–4 min 5% B, 4–38 min from 5% to 50% B (linear), 38–41 min from 50% to 90% B (linear), at a flow rate of 80 μL/min. MS/MS spectra were collected in data-dependent scan mode with a capillary temperature of 250°C, a source voltage of 3.6 kV and a capillary voltage of 40 V. Measurements were performed in positive ion mode and mass accuracy (FT) was calibrated before measurements. Ions were isolated with a width of 6–10 *m*/*z* and activated for 30 ms using 35% normalized collision energy and an activation q of 0.25.

HPLC-ESI-MS/MS data were analyzed by the Proteome Discoverer 1.2 program, based on the SEQUEST cluster as a search engine (University of Washington, licensed to Thermo Electron Corp., San Jose, CA) against the Swiss-Prot human proteome (released March 3, 2011; Swiss Prot human complete.fasta; 34,765 non-redundant protein sequences).

For matrix-assisted laser desorption ionization time-of-flight (MALDI-TOF)-MS, an Autoflex BruckerDaltonics apparatus was utilized. Samples were treated with a C-18 ZipTip pipette tip (Millipore) following the manufacturer’s instructions. The desalted solution was mixed 1:1 (v/v) with saturated solutions of R-cyano-4-hydroxycinnamic acid in acetonitrile/water (50:50, v/v) containing 0.1% TFA. Aliquots of 1 μL of the mixture were spotted onto the stainless steel target of the MALDI instrument. The calibration was performed using peptide calibration standards (angiotensin I and II, substance P, and bombesin, *m*/*z* range 1000–3150 Da). Positive MALDI spectra were acquired in either linear or reflectron mode with a pulsed nitrogen laser (337 nm). Mass spectra were acquired over the mass range of 700–6000 Da with a low mass cut-off of 500 Da and 400 scans were averaged for each spectrum.

### Statistical analysis

The mean and standard deviation analysis were calculated with Excel spreadsheet software (Microsoft Office 2010). The statistical significance was calculated with the Student’s t-test.

## Results

### Acetylation of AIRE NLS domain is required for the correct nuclear localization

It is well-established that lysine acetylation in the NLS domain regulates the distribution of several proteins. Starting from this evidence, we investigated the role of a set of lysines located in the NLS/N-terminal domain in the cytoplasmic/nuclear transport of AIRE. By the immunoprecipitation assay, described below, we first confirmed that AIRE is acetylated (data not shown), then, by MALDI-TOF-MS and RP-HPLC-ESI-MS/MS experiments, we mapped the acetylated lysine located in the AIRE protein.

To assess the role of endogenous acetyltransferase in the lysine acetylation of AIRE, we transfected COS-1 mammalian cells with pEF5-HA AIRE, in the presence of 10 mM sodium butyrate (NaBu), a potent inhibitor of histone deacetylases. Cell lysates were subjected to immunoprecipitation with an anti-HA antibody and, after enzymatic digestion, were analyzed by MS/MS.

**Figure 1:**
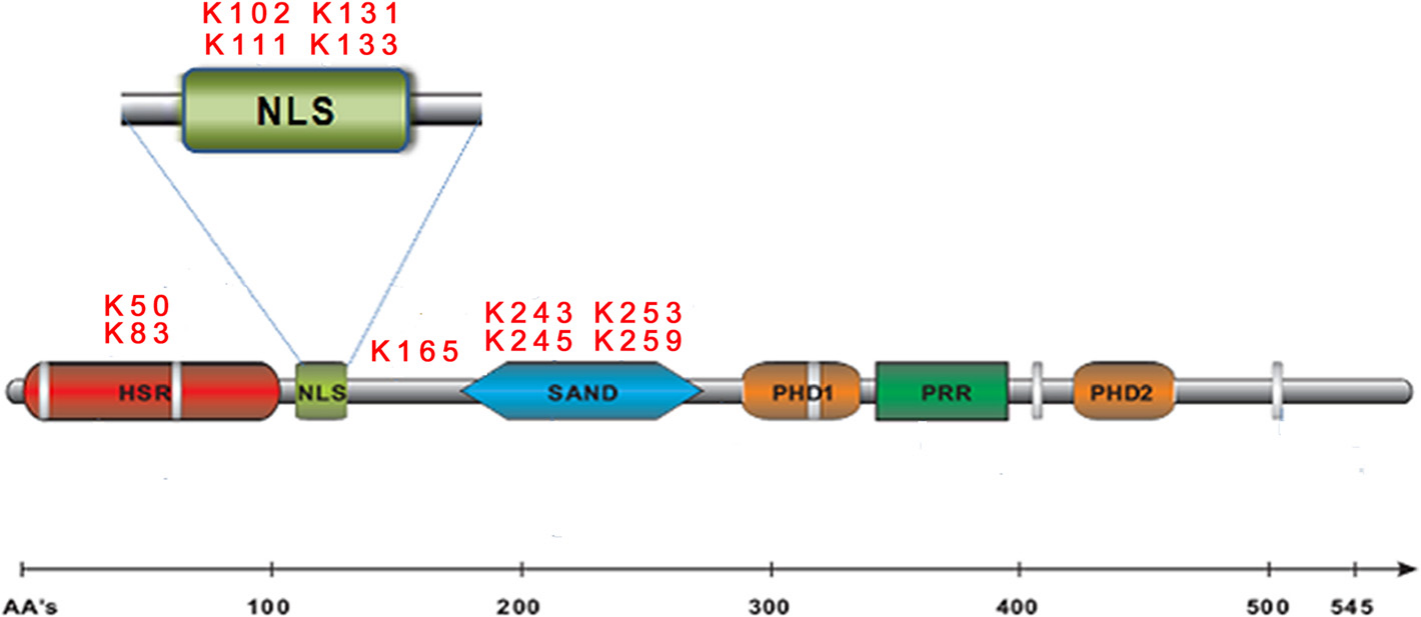
**Result of mass spectrometry of the 24 lysines of AIRE protein.** The acetylated lysines mapped by mass spectrometry experiments are represented. K: Lysine.

The 24 lysines scattered along the AIRE amino acid sequence, found by mass spectrometric analysis, are listed in Figure 1. A 42Da mass increase compatible with acetylation status was found in 11 out of the 24 lysines detected. This mass increase was not observed in AIRE extracted from negative control cells untreated with the deacetylase inhibitor NaBu (data not shown).

In contrast to data recently published by Saare et al**.** [12], we detected four acetylated lysines in the NLS/N-terminal domain at positions Lys102, Lys111, Lys131 and Lys133 and did not find acetylation at positions Lys114, Lys120, Lys159, Lys161 and Lys164. A possible explanation for this discrepancy is that our experiments were carried out in order to assess the role of the endogenous acetyltransferase. For this reason, no enzyme with this role, such as p300, was added.

The N terminus of AIRE contains two stretches of basic aminoacids (110-Arg-Lys-Gly-Arg-Lys-114 and 131-Lys-Arg-Lys-133) which were first predicted to function as a classical bipartite NLS [20]. Others studies have demonstrated that the N-terminus region of AIRE, from aminoacid position 101 to 141, is able to transport green fluorescent protein (GFP) into the nucleus, thus suggesting the presence of a functional NLS within this region [6]. Further mutagenesis studies [21] showed that residues 131–133 most likely function as a monopartite NLS that interacts with the minor binding site of importin-α family proteins. In these experiments, the authors showed that the presence of mutations in the N terminal part of the potential NLS (Arg113Ala, Lys114Glu or Arg113Ala, Lys114Ala) produced a punctate nuclear expression pattern similar to that of wild type AIRE, with only a minor decrease in the nuclear dot fraction. Conversely, a complete loss of NLS activity was observed in the presence of mutated residues at positions 131–133.

In contrast to these findings, Saltis et al.[18] claimed that a minor binding site is typically consistent with a bipartite NLS and that a monopartite NLS would preferentially bind to the importin-α major site. Furthermore, they observed that, in addition to the residues corresponding to human AIRE 131–133, NLS residues at positions 110–111 or 113–114 are also conserved in all species. Starting from these observations, Saltis theorized that this NLS (110–133) potentially functions as a bipartite NLS and that the residues at positions 110–114 are needed for the correct localization of AIRE in the nucleus [18]. However, at the moment, the exact mechanisms and the specific aminoacids involved in the nuclear localization of AIRE have not yet been completely established.

To investigate the role of the acetylated lysines found by spectrometry analysis, we carried out mutagenesis assays and fluorescence microscopy in order to verify if this post-translational modification can affect the subcellular localization of AIRE. AIRE cDNA was first cloned downstream of the green fluorescent protein (pGFP). Then, to investigate the effects of AIRE acetylation on molecular localization, we performed a set of site-direct mutagenesis experiments by introducing arginine, in place of lysine residues, to disrupt acetylation [22].

**Figure 2:**
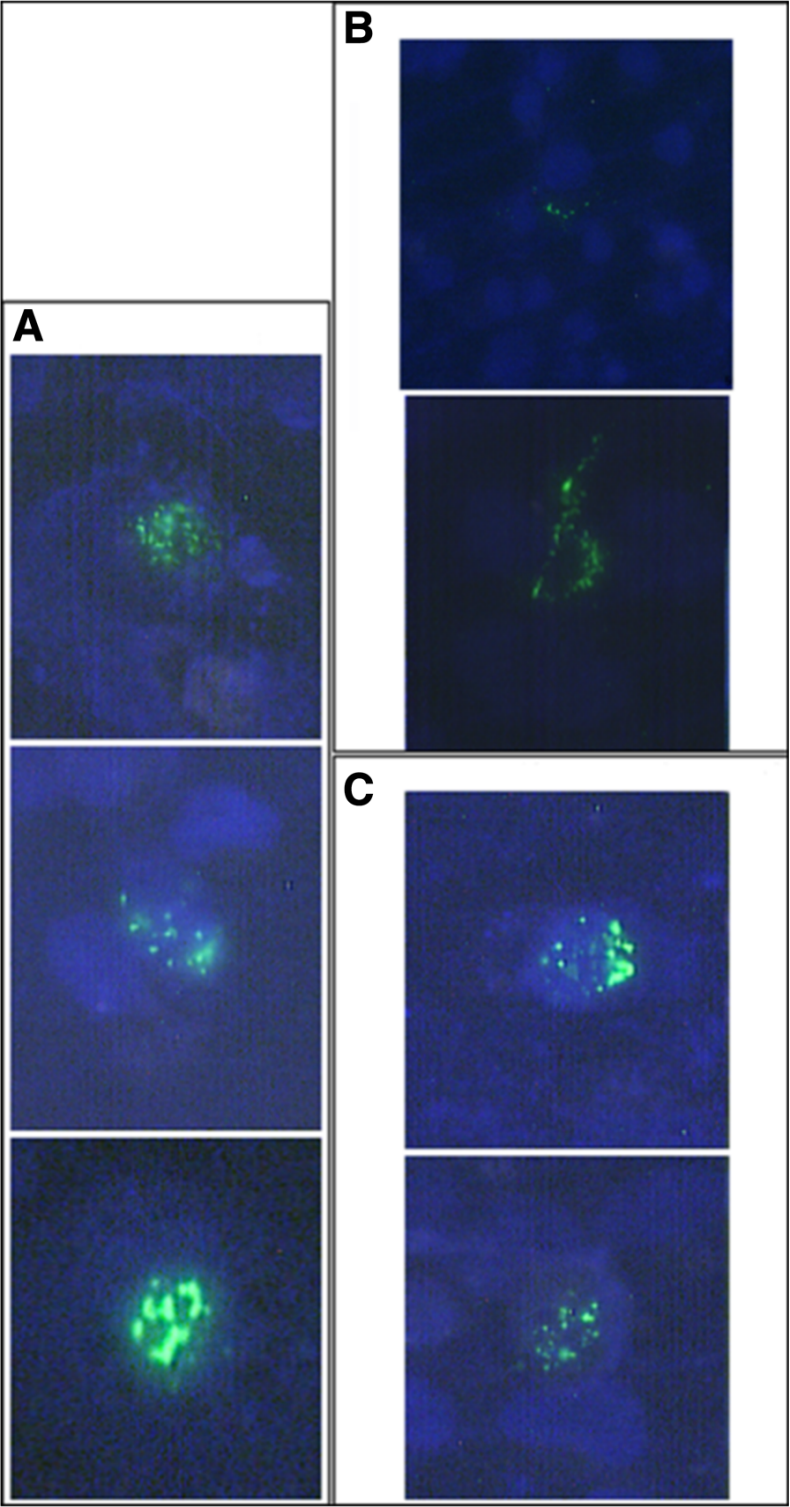
**Acetylation of AIRE influences the correct nuclear localization.** COS-1 cells were transfected with phMGFP–AIRE wild type **(panel A)**, phMGFP–AIRE Lys102Arg-Lys111Arg-Lys131Arg-Lys133Arg **(panel B)**, phMGFP–AIRE-Lys102Gln-Lys111Gln-Lys131Gln-Lys133Gln **(panel C)**. In contrast to the nuclear localization of wild type AIRE **(panel A)**, the quadruple Arginine mutant was diffused throughout the cytoplasm **(panel B)**. The wild type localization was restored in the quadruple Glutamine mutants **(panel C)**. The nuclei were stained blue with DAPI.

After transfection with GFP-AIRE wild type (wt) and GFP-AIRE Lys102Arg-Lys111Arg-Lys131Arg-Lys133Arg mutant constructs in COS-1 cells, we observed the nuclear dot pattern distribution by fluorescence microscopy. As shown in Figure 2, the GFP- AIRE wt construct exhibited a nuclear dot pattern (panel A), whereas the quadruple arginine substitution Lys102Arg-Lys111Arg-Lys131Arg-Lys133Arg exhibited a diffuse pattern and failed to produce dot-like nuclear staining (panel B). Correct nuclear localization was restored by replacing lysine residues at position 102, 111, 131 and 133 with glutamine residues which mimic constitutively acetylated lysines. Furthermore, as shown in panel C, nuclear transport was restored approximately as efficiently as wild-type AIRE.

In conclusion, these results demonstrate that AIRE is acetylated at positions 102, 111, 131 and 133 in the NLS domain and that modification can effectively influence AIRE localization in the nucleus.

This altered localization could be only partially attributed to aminoacid substitution, as both lysine and arginine are positively charged and share a similar structure. We rather believe that the different distribution of the protein could be due to the impossibility of arginine binding an acetyl group, thus confirming that acetylation is involved in the correct cellular positioning of AIRE. When lysine is acetylated, a condition mimicked by glutamine, AIRE may have a higher affinity for importins, a group of proteins which drives its nuclear translocation. However, we cannot exclude that the higher concentration of acetylated AIRE in the nucleus is due to reduced exportation. Indeed, AIRE contains a nuclear export signal (NES), within the HSR domain (aminoacids 1–100) and close to the NLS, whose functional regulation has not yet been completely clarified.

### AIRE is deacetylated

A second goal of our work was to determine if AIRE is deacetylated in vivo. In our previous work [23], we demonstrated that AIRE interacts with endogenous histone deacetylase 1 and histone deacetylase 2 (HDAC1, HDAC2), two targets for epigenetic repression that play an important role in transcriptional regulation through the deacetylation of histonic lysines. However, HDAC enzymes are not exclusively targeted towards histones. In fact, several non-histone proteins such as transcription factors, hormone receptors, signal transducers, chaperones and proteins of the cytoskeleton have been described to be subject to reversible deacetylation by HDACs [24]. The HDAC enzymes in eukaryotes are often associated with co-repressor complexes such as SIN3 and Mi-2/NuRD. In particular, the direct association of the SIN3complex (SIN3A HDAC1 and HDAC2) with a number of transcriptional regulators, causes the lysine deacetylation and is involved in the regulation of many essential cellular functions [25].

#### AIRE interacts with HDAC1-HDAC2/SIN3A complex

**Figure 3:**
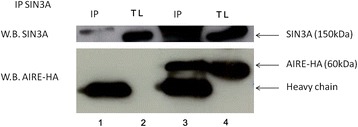
**Co-precipitation of endogenous SIN3A with HA-AIRE.** COS-1 cells, containing endogenous SIN3A, were transfected with plasmid pEF5HA-AIRE and immunoprecipitated using SIN3A antibody. (line 3 and 4). In line 1 and 2 the empty pEF5HA vector and its total lysate are shown. Upper panel: W.B anti SIN3A. Lower panel: W.B anti AIRE HA. W.B.: Western blotting. I.P.: Immunoprecipitated. T.L.: Total Lysate.

To investigate if AIRE interacts with the HDAC1-HDAC2/SIN3A complex, we first assessed the AIRE/SIN3A interaction. COS-1 cells, containing endogenous SIN3A, were transfected with plasmid AIRE-HA. Cell lysates were immunoprecipitated using an antibody against HA and then subjected to immunoblotting with an SIN3A-specific antibody. The results in Figure 3 show that endogenous SIN3A protein was immunoprecipitated with AIRE and demonstrate that there is likely a physical interaction between them.

#### AIRE is deacetylated in vitro

**Figure 4:**
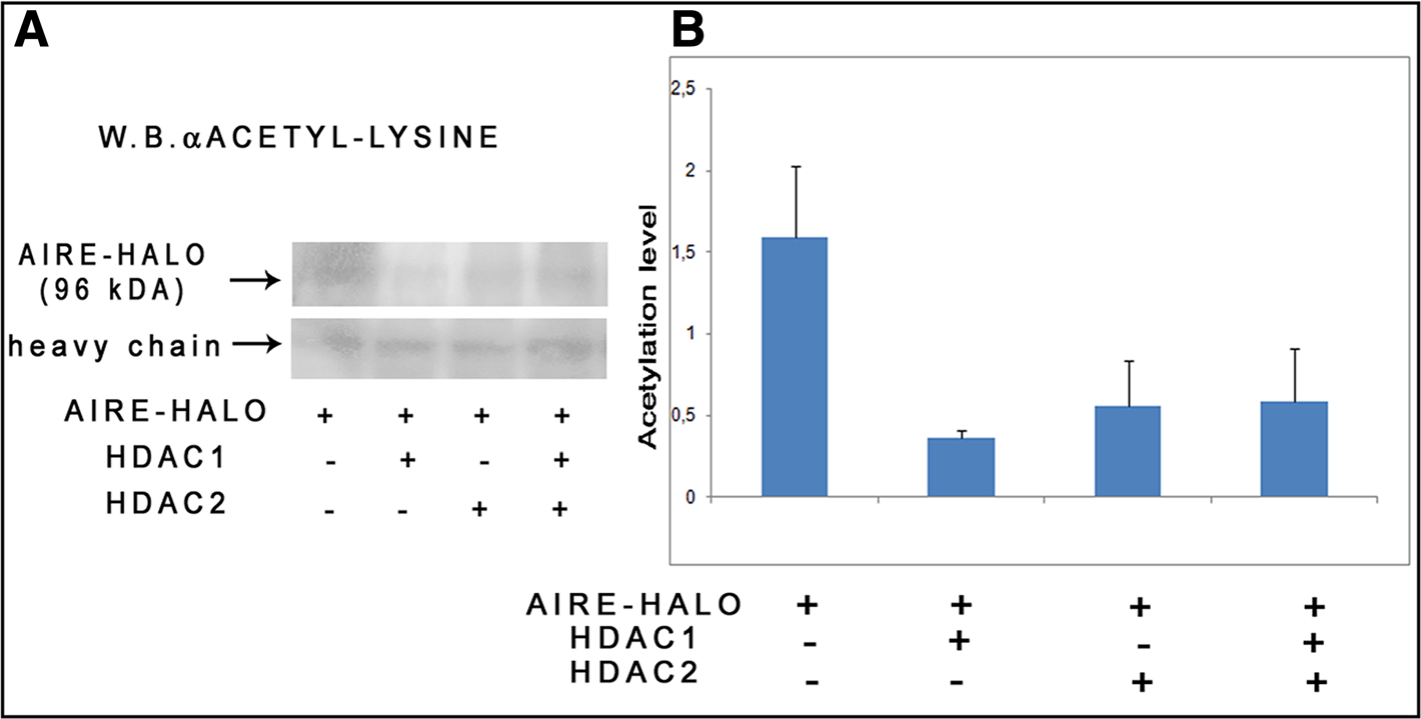
**AIRE is deacetylated by theHDAC1-2/SIN3A complex.** COS-1 cells were transfected with AIRE-HALO and/or HDAC1 and/or HDAC2 with endogenous SIN3A. Immunoprecipitation was performed on lysates using antibodies against HALO. Panel **A**: Anti-acetyl lysine signal of anti-HALO-AIRE immunoprecipitation. Panel **B**: The plot shows densitometric analysis using Image J. The acetylation level of AIRE was reduced by 71% in the presence of HDAC1 and 48% in the presence of HDAC2. Value are means ± SD (n = 2).

Subsequently, to test the hypothesis that the HDAC-SIN3A complex could be involved in AIRE deacetylation, we performed a deacetylation assay in COS-1 cells transfected with full-length AIRE-HALO, both in the presence and in absence of full-length HDACs, thereby taking advantage of endogenous SIN3A. Cells were immunoprecipitated using an antibody against HALO and then subjected to immunoblotting with an antibody against acetyl lysine (Figure 4 panel A). The level of acetylation was measured by a densitometry assay. The results show that the level of acetylation of AIRE was considerably decreased in the presence of the deacetylation complex. In particular, a 71% and 48% reduction of the level of acetylation, caused by HDAC1 and HDAC2 respectively, was observed. No cumulative effects were found when both HDACs were co-transfected with AIRE (Panel B).

#### HDACs repress the transactivation property of AIRE

**Figure 5:**
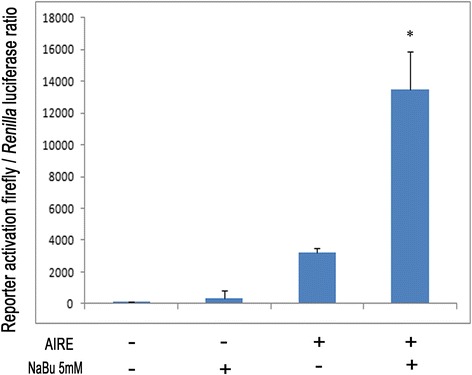
**The transactivation of AIRE is regulated by HDAC inhibition.** Values represent the relative luciferase activity (±SD) compared to the reporter plasmid transfected alone in the absence and in the presence of 5 mM NaBu. The ability of AIRE pbind -GAL4 to activate the gene reporter pGL5 was increased by four-fold in the presence of 5 mM NaBu. (n = 2), *P < 0.05.

Next, because HDACs are transcriptional co-regulators, we further investigated if the HDAC1-2 complex is involved in the regulation of AIRE transcriptional activity. To test this hypothesis, we performed a series of GAL4 system reporter assays in COS-1 cells. In particular, the full-length AIRE cDNA was expressed as a fusion protein with the GAL4 DNA binding domain, and the transcriptional activation was measured from a reporter plasmid containing a luciferase gene. First, we performed a transactivation assay of AIRE in the presence of NaBu, which inhibits HDAC activity. As shown in Figure 5, the presence of NaBu increases the transactivation activity of AIRE by four-fold, probably because it blocks the deacetylation activity of HDACs, thus leaving AIRE in a hyperacetylated status compatible with increased transcriptional activity (p < 0.05).

This finding is in contrast with the results on the role of lysines acetylation of AIRE protein recently published by Saare et al.[12]. They showed that the expression of mutant AIRE at positions Lys243 Gln,Lys245Gln and Lys253Gln, which mimic lysine acetylation, led to a 1.5- to 3.4-fold reduction in reporter activity, compared to wild type AIRE; furthermore, the negative effect of AIRE Glutamine mutant transcription of endogenous AIRE-regulated genes was even more drastic.

We suggest that the increased transactivation activity of AIRE, measured by transactivation assays with NaBu, may be due to the acetylation of lysines other than those placed in the SAND domain. In fact, previous studies have shown that other domains of AIRE, such as HSR, have transactivation activity [4], and the presence of lysines within this functional region could be a further target of acetylation. An intriguing hypothesis is that a dynamic interplay between acetylation and deacetylation of different domains may regulate the transactivation of AIRE.

**Figure 6:**
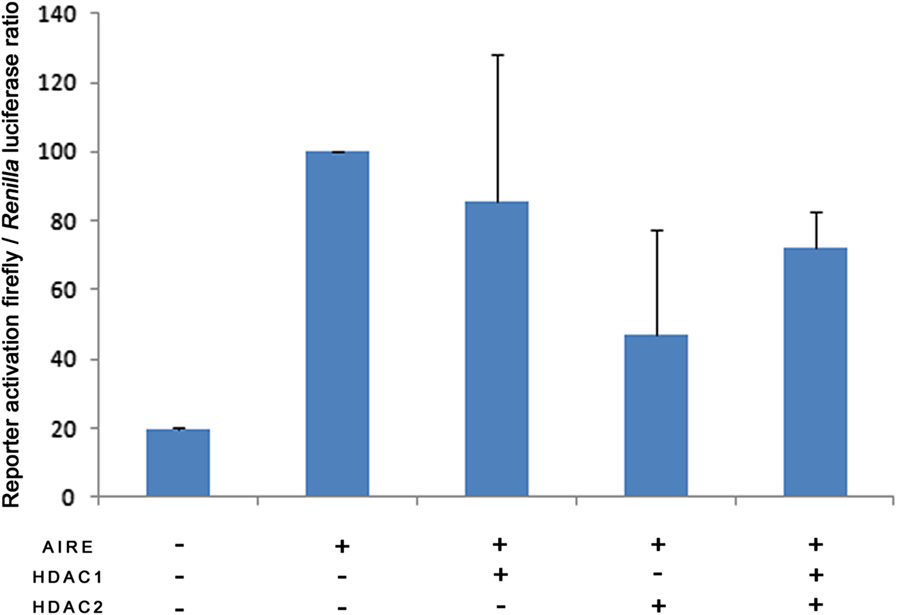
**The transactivation property of AIRE GAL4 is negatively regulated by HDACs.** The ability of AIRE pbind -GAL4 to activate the gene reporter pGL5 was decreased in the presence of HDACs. Values are means ± SD from at least three separate experiments.

Finally, we repeated the experiments by transfecting AIRE and HDAC (1–2) both jointly and separately. The results, shown in Figure 6, demonstrate that, in the presence of HDACs, the transactivation activity of AIRE decreased, again suggesting that the transactivation property of AIRE is modulated by the deacetylation activity of HDACs.

## Discussion

The AIRE protein contains several structural domains which are frequently found in transcriptional regulators [20,26], and are highly conserved in mouse [27-29]. Both endogenous AIRE and AIRE transiently expressed in mammalian cells are localized in nuclear bodies, known as ND10, which are tightly linked to the nuclear matrix and are among the most studied subcellular structures [7,9]. The integrity of nuclear dots can be impaired in severe diseases and their disruption has been found to be associated with the loss of translational control of a group of proteins, such as p53, Laf-1 and Daxx, which are tightly linked to ND10 [11,9]. When AIRE is transiently expressed in cultured cells, it localizes in the cytoplasm where it assembles in filaments, microtubules or aggregates of different sizes [30,31]. The N-terminus of AIRE, in particular the aminoacid residues at positions 101–141, are able to direct the GFP protein into the nucleus, thus suggesting the presence of an NLS within this region [6]. A comparative analysis of AIRE [18] has suggested the existence of a bipartite NLS extending from lysine 110 to lysine 133 and a second putative NLS between lysines 157 and 159. Moreover, a complete loss of NLS activity has been observed in the presence of mutated residues at positions 131–133 [21]. Lysines are essential components of NLS and are often acetylated. It has been shown that acetylation enhances nuclear/cytoplasmic shuttling of several proteins and, in some cases, it is essential for this dynamic action. For example, POP-1 requires acetylation of certain lysines to gain entry to the nuclear compartment, whereas acetylation of proteins such as Max, c-Abl and RECQL4 increases their cytoplasmic localization. It has been proposed that lysine acetylation can regulate NLS function by affecting NLS interactions with the nuclear import machinery. Alternatively, acetylation within the NLS could induce conformational changes that affect the NLS or nuclear export signal functions and alter protein subcellular distribution, as described for several proteins.

Our results indicate that lysine acetylation in the N terminal region of AIRE containing the NLS domain is important for its subcellular localization. By MALDI-TOF experiments, we identified four acetylated sites (Lys102, Lys111, Lys131 and Lys133) that are involved both in AIRE nuclear retention and in its regulatory function.

In fact, we have shown that the lack of AIRE acetylation in mutated lysines (Lys102-111-131-133Arg) implies a reduction in nuclear import and that Lys102-111-131-133Gln substitutions, which mimic the presence of acetylated lysines, recover the correct nuclear localization.

Starting from our previous results demonstrating that AIRE directly interacts with HDAC1/HDAC2 deacetylases, we speculated that AIRE is deacetylated by the HDACs/SIN3A complex. Moreover, we reported that NaBu, an inhibitor of histone deacetylases (HDACs), enhanced the ability of AIRE to promote transcription of an insulin reporter gene. Consistent with this finding, we demonstrated that, when AIRE physically associates with HDACs/SIN3A in vivo, its transcriptional activity decreased. We hypothesize that the interaction is not direct, as for other proteins (p53) but, rather, mediated by the co-repressor SIN3A. One of the emerging concepts is that acetylation status regulates protein stability. Lysine residues are targets for both acetylation and ubiquitination, and one of these modifications seems to prevent the other. Deacetylation by HDACs is, in many cases, a prerequisite for subsequent ubiquitination. Conversely, acetylation may protect a protein from ubiquitination and degradation.

## Conclusions

**Figure 7:**
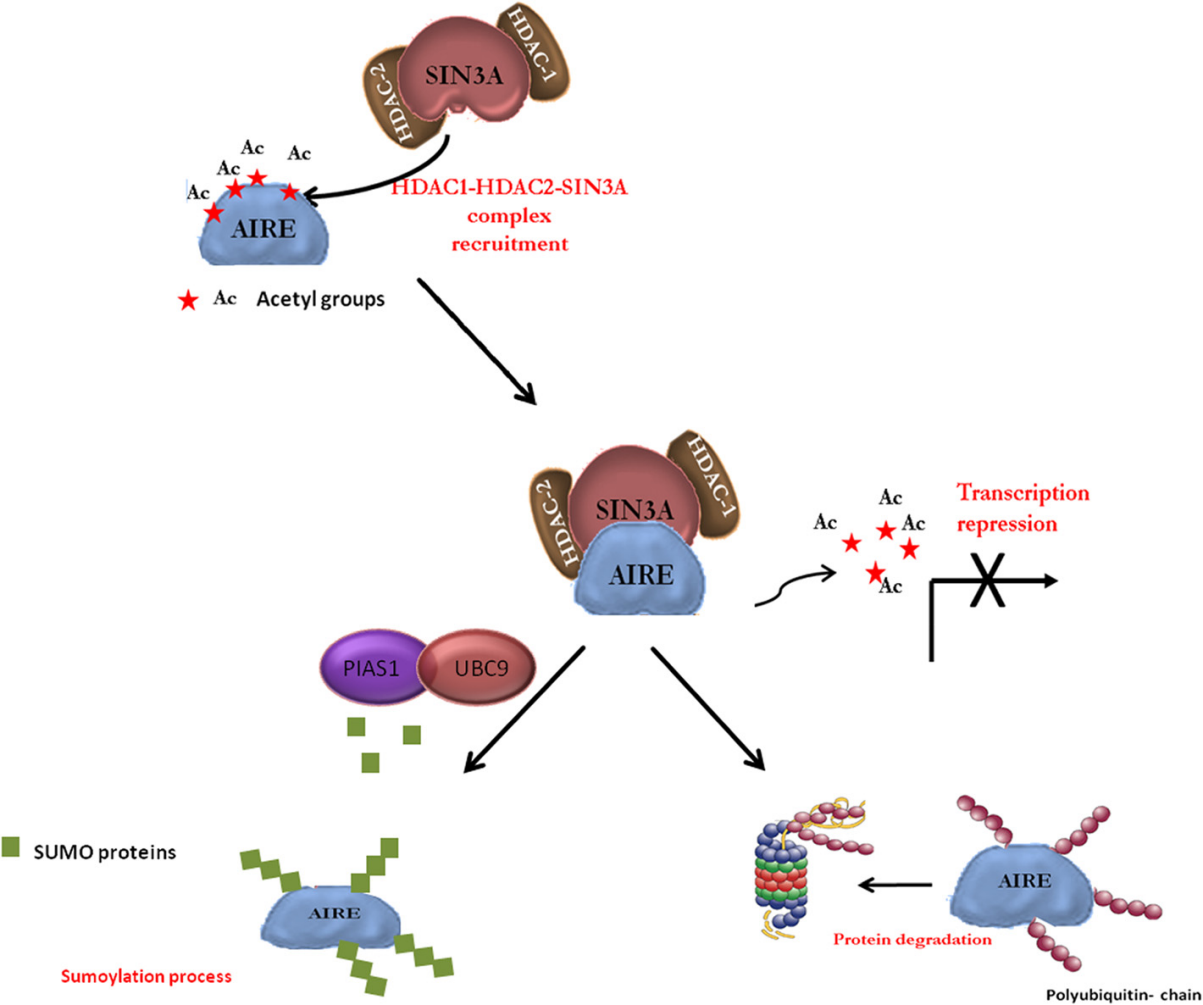
**Hypothetical model of the roles of HAT and HDAC in the regulation of AIRE protein.**

In conclusion, we observed that the interaction of AIRE with deacetylases complexes causes the removal of acetyl group from lysines, thus inhibiting the transcriptional activity of AIRE (Figure 7). This deacetylation status is probably responsible for the instability of AIRE, which becomes more susceptible to degradation in the proteasome. In fact, as previously stated [11] AIRE functions both as an E3 ubiquitin ligase and is also subject to ubiquitination. Interestingly, the authors have also found that proteasome inhibitors increase the number of AIRE-containing nuclear bodies, suggesting that ubiquitination plays a role in regulating AIRE localization. Thus, acetylation status could regulate the stability of AIRE, which in this manner could escape ubiquitination and proteasomal degradation.

Future investigations will seek a better understanding of other post-translational modifications of AIRE which could possibly be involved in the correct localization or in the regulation of its transcriptional activity. For example, we believe that the role of sumoylation could be further investigated in this direction. In previous unpublished experiments, we characterized Ubc9, an E2 SUMO enzyme, as being a partner AIRE, and Ilmarinen et al. [32] have demonstrated that AIRE binds PIAS1, an E3 SUMO ligase. As lysines can be targets for sumoylation and because sumoylation is often opposed to ubiquitination, it would be of interest to investigate the role of other lysine modifications in the correct functioning of AIRE. These studies would give a better comprehension of the molecular pathogenesis of APECED and would also serve as a model for other autoimmune diseases. The definition of the role of the AIRE protein could also lead to the development of an appropriate gene therapy and also to the development of targeted therapeutic approaches.

## Abbreviations

K: Lysine
R: Arginine
Q: Glutamine
